# The North Pole

**Published:** 1867-08

**Authors:** 


### THE NORTH POLE.

Considering the value of food and the comfort of keeping it in a fresh condition, a philosophically constructed refrigerator becomes one of the most economical and comfort-giving articles of household use. That is beyond all question the best Refrigerator which combines in one, the indispensible advantages of light, cleanliness and cold.

The "Zero Refrigerator" shows at a single glance every article of food in it, being lined with zinc it is easily kept clean; the ice being put in at the top; and the provisions through a front opening, the several advantages are secured of, First, no grease, crumbs, bits of meat, or berries get mixed with the ice. Second, as cold air settles at the bottom, the coldest part of the Refrigerator is that which, being under the ice, contains the food. Third. As the ice melts, the water which it makes is conveyed into a reservoir, and is delivered by a faucet, so that there is always ice-cold water at hand.

The prices vary according to size — from $25 to $50. Sold by the Patentee, Alexander M. Lesley, 605 Sixth Avenue, and 1310 Broadway, New York. Among the purchasers who give high praise to this most perfect of all Refrigerators, to this date of July 4, 1867, are

| | |
|---|---|
| F. S. Winston, of New York. | John Caswell, New York. |
| Pitt Cooke, of Jay Cooke & Co., N. Y. | C. R. Topliff, of Munn & Co., N. Y. |
| E. H. R. Lyman, Brooklyn. | Judge Storer, Cincinnati, Ohio. |
| Hon. Erastus Brooks. | Hon. James Brooks. |
| Mrs. Sequin, Staten Island. | F. Schuchardt, New Hamburgh, N. Y |
| Josiah Mann, New York. | John Morrison, New York. |
| J. R. Briggs, Sheffield, Mass. | O. de Comeau, Spuyten Deyvil. |
| W. H. Butler, of Valentine & Butler. | H. D. Oliphant, Orange, N. J. |
| I. M. Morrison. | Richard B. Kimball. |
| F. B. Nichol, of Houghwout & Co. | H. A. Tilden, New Lebanon, N. Y. |
| Wm. B. Green, Newport, R. I. | Edward Sweet, Montclair, N. J. |
| L. Waefelaer, New York. | W. Whitlock, Jr., New York. |
| J. H. Sherwood, " | Wm. H. Lee, " |
| Hon. Geo. Sthephenson, New York. | Rev. J. P. White, Newport, R. I. |
| Rev. A. S. Twomley, Stamford, Ct. | Chas. A. Clayton, Orange, N. J. |
| Jacob Hays, Inwood, N. J. | Charles Curtis, New York. |
| R. B. Henchman, Cincinnati, Ohio. | G. L. Weaver, Albany, New York. |
| Sailors' Snug Harbor, Staten Island. | Rabbi Isaacs, New York. |
| Wm. Kinsey, Greenwich, Ct. | J. F. Kendall, New York. |

Orange Judd, of the "American Agriculturist."

J. J. Thomas, of the "Cultivator and Country Gentleman."

N. P. Boyner, of the "American Stock Journal," and hundreds of others.

